# Quality requirements for cross-border care in Europe: a qualitative study of patients’, professionals’ and healthcare financiers’ views

**DOI:** 10.1136/qshc.2008.028837

**Published:** 2009-01-26

**Authors:** O Groene, P Poletti, P Vallejo, C Cucic, N Klazinga, R Suñol

**Affiliations:** 1 Avedis Donabedian Institute, Autonomous University of Barcelona, and CIBER Epidemiology and Public Health (CIBERESP), Barcelona, Spain; 2 Centre for Research and Development, Padua, Italy; 3 Avedis Donabedian Institute, Madrid Office, Autonomous University of Barcelona, and CIBER Epidemiology and Public Health (CIBERESP), Barcelona, Spain; 4 The Dutch Institute for Health Care Improvement, the Netherlands; 5 University of Amsterdam, Amsterdam, the Netherlands

## Abstract

**Background:**

In the past decade the issue of patient mobility has emerged on the European health policy agenda. Although the volume of patients crossing borders to obtain healthcare is low, it is increasing continuously and, due to its legal, financial and medical implications, has generated considerable interest among health policy and other decision makers. However, there is little information available on the safety and patient-centredness of cross-border care and neither governments nor citizens have an explicit basis for comparing healthcare delivery in Europe.

**Methods:**

This study investigated the viewpoints of patients, professionals and healthcare financiers on the safety and patient-centredness of cross-border care. Qualitative interviews were carried out during 2005 and early 2006 with 40 patients, 30 professionals (doctors, nurses and managers) and 3 healthcare-financing bodies.

**Results:**

Although cross-border care has become a common issue in many European countries, there remain uncertainties on the side of each of the parties addressed—patients, professionals and financiers—with regard to the provision of cross-border care. One of the most striking results of this project is the current lack of research on systematic knowledge on the quality of cross-border care.

**Conclusion:**

Many of the issues identified through this research may have a potential impact on the quality and safety of cross-border care and will support further investigation and help shape the health policy agenda on patients crossing borders in European Union countries.

The provision of cross-border care is a phenomenon that received considerable policy attention in past years. In response to this phenomenon, and considering a range of unresolved legal, financial and administrative issues, and the implications for patient care, the European Commission (EC) financed a number of “Scientific Support to Policies” (SSP) activities. The research presented in this paper was conducted within the “Methods of Assessing Response to Quality Improvement Strategies” (MARQuIS) Project, one of the EC-funded SSP projects. The legal framework under which cross-border care takes place, the general framework for the SSP policies and an introduction to the background and objectives of the MARQuIS Project are presented elsewhere in detail.^1^ ^2^

There are different reasons for patients seeking or obtaining care in a different country: visitors abroad who might need of emergency care; retired people moving as long-term residents to other countries need the same network of general and specialist services as local patients but with different quality requirements; people living in border areas may cross borders to circumvent waiting lists; people may be referred abroad for specialist services; and some people seeking treatment abroad themselves.^3^ Each group of patients may have different expectations, quality requirements and a different experience of the care process.

An extensive literature base exists on assessing and improving patient safety and patient-centredness of hospital services.^4^ ^5^ In addition, there is growing literature on the expectations and quality requirements for groups of patients who have limited literacy skills, patients who do not speak the local language, immigrant patients or patients who differ in their cultural views and expectations.^6^^–^10

However, knowledge about patient safety and patient-centredness issues related to patients crossing borders to obtain care has remained rather scarce until now.^11^ Studies have found that there is remarkably little systematic information on the volume and scope of cross-border care, and in particular on the perspectives and outcomes of cross-border care at the patient level.^12^ ^13^ Some surveys on experiences of cross-border patients indicate that this group of patients encounters problems regarding travelling (financially and logistically), emotional issues associated with distance from home, unfamiliarity with access procedures and continuity of care.^14^

The aim of this study was to assess whether particular quality requirements exist for this group of patients. We focused our study on patient-centredness and safety issues because of their transversal nature and relevance to cross-border care. These terms were defined as follows: “Patient-centredness refers to health care that establishes a partnership among practitioners, patients, and their families (when appropriate) to ensure that decisions respect patients’ wants, needs, and preferences and that patients have the education and support they need to make decisions and participate in their own care”,^15^ while “patient safety refers to the prevention of harm caused by errors of commission and omission”.^16^

Taking into consideration patients’, professionals’ and healthcare financiers’ experiences, the research presented in this article led to the identification of potential safety and patient-centredness issues for which further research, the development of instruments and quality improvement actions might be indicated.

## MATERIAL AND METHODS

Qualitative interviews were carried out to collect information on the views of patients, professionals and financiers on the safety and patient-centredness of cross-border care. For our study we considered all types of cross-border patient as one group, although interviews with professionals allowed identification of some topics associated with a particular group of cross-border patients. To guide the identification of issues, we used a simplified service delivery model reflecting a functional perspective of hospital services from the patients’ perspective:^17^

- “Patient admission” entails information, activities, processes and systems that are related to administrative requirements and access to care for both emergency or planned admissions.
- “Diagnosis and intervention” comprises all activities related to direct care and the treatment of patients.
- “Discharge” embraces information and systems related to the end of the care process at a given location, transfers to another provider or patient support to go back home.

A semi-structured interview guideline was developed based on the conceptual model and existing literature on cross-border care issues, which was revised after carrying out a pilot test (interview guidelines are available on request from the first author). Interviews were structured in three phases: introduction, topic generation and closure.^18^ ^19^ The introduction phase aimed at clarifying the objectives of the interview and identifying the day-to-day experience the interviewee had with cross-border care. The topic generation phase aimed at generating knowledge around the experience of the interviewee with cross-border care issues. Prompts were used to elicit feedback when the interviewee found it difficult to respond to a question. In the closing phase, the main views of the interviewee with regard to patient safety and centredness issues for cross-border care were summarised to double-check understanding.

### Participants

Due to the relatively low volume of cross-border care, random sampling would have been unfeasible. Therefore, patients, professionals and financiers were identified by convenience sample across seven countries participating in the MARQuIS project. Recruitment of additional interviewees was stopped when saturation was reached. For patient interviews, contact persons in the participating hospitals identified eligible interviewees and obtained informed consent to participate in the interview, which was carried out shortly before discharge. Interviews were conducted in five languages. The professionals’ perspective was studied by interviewing managers and health professionals (doctors and nurses) from facilities with a high volume of cross-border patients. In each of the study hospitals, the chief executive officer (CEO) identified a manager, a doctor and a nurse with experience in delivering cross-border care, for participation in the study. Professionals’ interviews were conducted in three languages. For the healthcare financing perspective, interviews were carried out with representatives from major European health financing institutions, based in the Netherlands and in Belgium, with experience in purchasing cross-border care.

### Data collection and analysis

Interviews were held in person (for the patients’ and the financiers’ perspective) or on the phone (for the professionals’ perspective). Interviews lasted 25–45 min and were recorded and transcribed. A member of the research team (the main author) reviewed the interview transcripts and notes for themes and emergent issues and tagged them manually.^20^ In a first step, the results of the interviews carried out with patients, professionals and healthcare financing bodies were summarised independently. In a second step, the views of patients, professionals and financiers on the safety and patient-centredness of cross-border care were assessed for convergent or divergent responses. Interviews were carried out during 2005 and early 2006.

## RESULTS

Overall, 40 EU patients from 14 countries admitted to 7 different hospitals in Italy and Spain, 30 health professionals from 14 hospitals in 6 European countries (Belgium, Czech Republic, Ireland, Poland, Spain and the Netherlands) and interviewees from 3 healthcare financing bodies (in Belgium and the Netherlands) participated in this study. We analysed the safety and patient-centredness issues for cross-border care across the three groups of interviewees and four transversal themes emerged from this analysis:

- communication and information issues;
- administrative procedures;
- clinical procedures;
- hotel services and physical environment.

Boxes 1–4 illustrate some of the statements of the interviewees that led to the identification of themes. Due to the large amount of information we have not reported all direct statements of interviewees; instead we have summarised the main research and improvement topics that we derived from these statements in appendix A.

**Box 1: Example statements for communication and information issues**

“There are a number of difficulties in assessing [cross-border] patients, for example, that the patient does not carry required information/clinical record, or brings medication that can not be clearly identified so it is difficult to know what to provide. Many patients come alone and this brings along further difficulties as there is no one to provide answers. There are obvious problems in evaluating symptoms if the patient does not speak English.” (Professional PRO-1)

“[cross-border patients] are usually more critical and have higher information requirements, but in the end they are even more satisfied than local patients because they compare the care they received with what they would have got in their own country.” (Professional PRO-9)

“a pregnant woman … could not speak any language apart from her mother tongue, [she] did not understand what was going on to her, and the woman not understanding the basic rules of hospitalisation like switching off of mobile phone in the night and visiting hours, this caused some problems to the other patients in the room” (Professional PRO-3)

“Three different interpreters went around the unit asking if there are problems, but they are not there when needed, they are more there to listen rather than really help. I was not asked what my needs were” (Patient ES7-IRL-M-2)

“In the emergency department there was too much chaos, too many people, and I was suffering. I think that there was a ‘privacy problem’ since doors were kept open and everybody passes by … it would be better to keep the doors closed. There is too much noise at the hospital. It is difficult to speak. Nurses would open the door while they [patients] are sleeping, making a lot of noise.” (Patient ES15-F-M-2)

“The reality is that it [good communication] is not ensured; for example, language problems can cause problems in cross-border care. There are regions where there’s more experience with cross-border care and they work it out in the region. It’s important to discuss cross-border care at European level, because a lot can be learned from each other.” (Financier FIN-2)

**Box 2: Example statements for administrative procedures**

“Before the patient arrives I have had contact already, they send file, I need to agree, everything is managed before, the patients that are referred to me never come by hazard. Patients need the E-112, but sometimes the country is reluctant—there is no free movement of patients. [Movement of patients] should not only be administrative process, there should be recommendations by [the specialist] to provide surgery” (Professional PRO-6)

“Sometimes there may be problems to discharge patients. … Some patients need to stay in a hospital but not in this one and then sometimes it is difficult to find an ambulance to transport the patient to his country, especially on Fridays.” (Professional PRO-1)

“A serious issue is the organisation of back-transfer. We needed to transfer a patient [across the border] and when he arrived [at the hospital], there was no ICU [intensive care unit] bed available. They brought him back to our hospital, but he died on the way. There is lack of information systems to check the availability of beds.” (Professional PRO-22)

“We always visit hospitals we want to contract to have an impression at least of how the care is in the hospital.” (Financier FIN1)

“Now, a special issue for quality of cross-border care is the combination of quality aspects from both the home and the host country …. For the quality of care abroad you must rely on regulatory bodies or ministries or legal frameworks concerning quality of that country. But the combination, that may be the problem. Especially in cases where follow-up treatment is given in the home country of the patient.” (Financier FIN3)

“There was miscommunication between the insurance and the hospital, missing faxes, dealing with the paperwork was terrible… I had to advance the money…” (Patient ES16-NL-F-2)

“It is very difficult for the patient to find information on what they have to expect on the other side of the border. There’s no regular information or regular basis to find this kind of information, no books, brochures on this. We are working on providing patients with good information, for instance on a … healthcare portal with that information.” (Financier FIN3)

**Box 3: Example statements for clinical issues**

“Differences exist in type, name and dosage of drugs; this is a potential patient safety issue.” (Professional PRO-5)

“In our hospital we have more than 200 consent forms, depending on the pathology and we considered translating them, but of course, with so many forms that need to be updated, this is not feasible.” (Professional PRO-1)

“In Germany it’s legally arranged: informed consent. In Belgium it’s more like the Dutch situation: the doctor will tell you [verbally] all the possible complications of the treatment. So an important aspect would be an informed consent written in your own language. That’s not the case at present. In Germany you get an informed consent written in German.” (Financier FIN-2)

“One important example [for differences in clinical procedures] would be transplantation medicine. For kidney transplants the waiting list is very long, but we have found a way to use kidneys from non-heart-beating patients. Although there are some minor differences in the quality of the organs, we have achieved some really good results. I have now experienced that it is forbidden in Germany to use organs from non-heart-beating patients and I have tried to introduce to my German colleagues our experiences. But there was a major reaction: ‘This will never be done in Germany!’. The response was that this is never going to happen in Germany, there seem to be major cultural differences with regard to the use of organs from non-heart-beating patients.” (Professional PRO-14)

“Many foreign patients set a time limit for the in-patient stay ‘I can stay until Sunday, then I have to take my plane!’. Even if we tell them that it is not safe, they sometimes insist. There is a form for voluntary discharge to be signed.” (Professional PRO-15)

**Box 4: Example statements for hotel services and physical structure**

“Patients from Nordic countries that are used to quiet environments and everything being in order are sometimes scared when they arrive at our hospital because everything is much more busy, noisy and does not seem to be so organised to the Nordic patients. Also, the times when meals are served and our working times are strange to these patients. Patients from other Mediterranean countries are closer to our culture, for them there is no major difference.” (Professional PRO-10)

“The bed was extremely squeaky and with the noise she made she disturbed me. When I asked the doctor for another bed, he said that I was in a hospital, not in a hotel. The communication is terrible, and food is not nice, and water is not enough, and the smell of the detergent they use for personal care is unpleasant” (Patient ES2-D-F-2)

“When he [the husband] arrived at the hospital he had to be reanimated. They put me in a waiting room and did not provide any information. I did not know what was going on. Only after everything was over someone informed me about what had happened. When the emergency was over, the hospital called the hotel and even British Airways.” (Patient ES17-UK-M-1)

Cross-border patients have communication and information needs at all stages of the care process—admission, diagnosis/intervention and discharge—and their needs are not limited to clinical issues but rather address the whole healthcare process as well as the scheduling of tests and procedures for which communication is very important. Differences exist in the way patients collaborate and get involved in decisions about their own treatment, including the role of the family in different cultures. Patients found it difficult to identify the different professional profiles (other than the doctors) and requested further information in different languages on the hospital and unit organisation. Professionals confirmed the lack of communication between providers for cross-border patients—for example, contacts with the family doctor to validate the history of the patient, during both the admission and discharge phases (box 1).

All three groups of interviewees confirmed that administrative requirements regarding forms E111 and E112 can pose problems in individual cases, such as when patients do not have the forms or use them fraudulently. Patients were not worried about hospitalisation charges; however, financiers and professionals consider cross-border patients to be more expensive because of the higher administrative burden and information requirements. Health financiers mentioned that they would like to selectively contract with individual providers whereas professionals think that contracts should be made at the health system level and not with individual providers.

With regard to the authorisation to obtain elective treatment abroad, there seem to be cases where free movement of patients is not fully ensured and interviewees pointed out that eligibility needs to be assessed more strongly based on recommendations by specialists, not solely on administrative grounds (box 2).

The issue of informed consent relates both to communication needs and to administrative and clinical procedures. In many cases, informed consent forms are only available in the local language (due to legislation on medical documentation) while in others a variety of forms was translated into foreign languages. The extent of informed consent also varies from a form simply declaring that the patient agrees with performing a procedure to a comprehensive patient information package that requires the patient’s signature. Similar legal issues and differences in scope relate to the information provided on discharge.

Differences in medical procedures have been found between countries with regard to transplantation procedures (related to the use of organs from non-heart-beating patients), quantity of rehabilitation services or caesarean section on demand (which is not uncommon in some countries). A particular problem for cross-border patients is that discharge date (either too early, or after excessive length of stay) may be influenced by previously made travel arrangements, rather than medical criteria.

Patients may also have problems obtaining medication from local pharmacies, particularly if they have different names or dosages (box 3). Patients, professionals and financiers confirmed that cross-border patients make higher demands on a hospital’s hotel services. The most common requests related to issues such as timing and size of meals and privacy; other main issues were respect for families’ or carers’ needs, accommodation, information about the patient’s situation and purchasable items. Professionals pointed out that cultural differences exist in the way patients from the north, south and east of Europe collaborated in the care process (box 4).

## DISCUSSION

The research base for the quality of cross-border care is currently not very well developed. Our research helped to identify some issues related to patient safety and patient-centredness of cross-border care that may inform further research and quality improvement activities.^21^ In general, although patients express positive opinions regarding the quality of care they receive abroad and professionals claim that they treat local and cross-border patients alike, there are some potential quality and safety issues for cross-border care, because of the information requirements and complexity of the care process, especially when follow-up care is required.

### Information and communication issues

Information and communication were the main priorities of cross-border patients, irrespective of the reason for admission. These priorities addressed the whole hospitalisation from admission to discharge. There is substantial literature evidence that information and communication are integral components of the care process;^22^ ^23^ in fact, full access to healthcare includes cultural and linguistic access as well as financial access.^24^ Some hospitals employ a range of strategies to address this issue, such as multilingual reception, written information in various languages and/or professionals (such as but not limited to interpreters) speaking various languages. For cross-border patients, families and carers also play an important role in ensuring information exchange.^25^ Interviews with patients and professionals also showed that some cultural differences seem to exist regarding the patients’ expectations and their ability and willingness to collaborate in the care process: patients from some countries were found to be particularly critical, whereas others were considered to be more passive in the care process.^26^ The discharge and follow-up phases appear to be among the weakest points in the cross-border care process. Ensuring continuity of care will remain a challenge in the absence of common requirements for hospitals in Europe concerning the type and timeliness of information provided on discharge to the patient and to health services providers. All groups of interviewees confirmed this issue, which is also supported by the findings of a parallel study.^27^

### Administrative procedures

For patients who intentionally travel to another country to obtain care, a clear understanding of administrative procedures is a prerequisite to making an informed decision. Given the fact that cross-border care is a growing phenomenon in Europe,^1^ ^28^ the lack of information sources on treatment in different European countries is worth highlighting. On the other hand, patients who unexpectedly need treatment while abroad commonly face the problem of not having the needed documentation and not being familiar with bureaucratic procedures. As a result, patients get hospitalised without the E111 form, which they usually apply for only once they are at the hospital. This bureaucratic problem can cause unnecessary delays in care. In particular, patients with chronic health conditions, and those with recurrent acute health problems, who plan to travel should be provided with relevant information about their disease or condition, to avoid repetition of tests and dangerous or unnecessary delays.

### Clinical procedures

There appears to be wide discrepancies among European Union (EU) member states regarding the process of providing informed consent. Even if some countries have translated the forms into some EU languages, they are often not translated into all the needed languages and there is no systematic procedure for informing patients. This has implications, in particular, for the comprehensive informed consent procedures required for clinical trials, as the lack of forms for providing informed consent in different languages currently makes it difficult to include cross-border patients.

We also identified potential safety issues with regard to discharge date and back transfer (in particular with regard to contracting ambulances to travel to a different country or arranging a hospital bed in another country). Other safety issues involve quarantine procedures for methicillin-resistant *Staphylococcus aureus* (MRSA).^27^ Another potential patient safety issue is related to drug use, as drugs may be prescribed and/or administered that have different names or dosages in other countries. Common requirements for information provided at discharge and generic name prescribing may improve cross-border care. However, the development of integrated care networks even faces challenges when professional and organisational boundaries within countries need to be crossed, and standardisation of criteria, protocols and rules cannot substitute for direct contact to share information and knowledge.^29^

**Key messages: How this paper links to better patient care**

This study identified a range of issues that potentially impact on the quality and safety of cross-border care. While quality requirements for cross-border care are similar to those of local patients, certain situations exist that require particular attention to the needs of cross-border patients. These situations involve, for example: communication problems related to history taking and informed consent; medication safety at discharge due to differences in type and dosage of drugs; ensuring continuity of care after discharge due to lack of information provision to patients and subsequent healthcare providers; and difficulties in arranging medical back transfer of patients across borders.

### Physical structure and hotel services

Regarding hotel services, there are indications that cross-border patients have higher demands, mostly related to privacy and the possibility of purchasing needed items. Cultural differences in family involvement (among other aspects) may pose further challenges to hospital organisation, and hospitals providing cross-border care for elective procedures may need to assess how to accommodate patients and families before and during admission.

### Limitations of the research

It needs to be emphasised that the legal framework under which cross-border care is provided may have implications for administrative and quality requirements. For example, countries with bilateral contracts may have arrangements in place to regulate volume, tariffs, procedures, quality and non-medical aspects of hospital care, and may hence avoid some of the issues related to cross-border care provision identified in this research. Thus, the exploratory nature of this research deserves particular emphasis given different contexts of cross-border care provision.

From a methodological perspective a number of limitations need to be stressed. First, interviewees were recruited through convenience sampling in order to include as much variation as possible. Only two Mediterranean countries participated in the interview arm of the study—one of them a major exporter of patients (Italy) and another a major receptor (Spain). However, views were collected for patients from 14 countries. Healthcare professionals were recruited from hospitals known to deliver cross-border care. The interviews with healthcare financiers were conducted with two financing bodies in Belgium and the Netherlands, with one interviewee representing an umbrella organisation of healthcare-financing bodies. While this sampling method can be criticised and is likely to have induced significant bias, it should be kept in mind that random sampling would not have been feasible due to the low frequency of cross-border care. Thus the results of this research should not be misunderstood to convey a generalisable picture of cross-border care provision. Rather, the strength of this research is that, given the current dearth of information on quality requirements for cross-border care, the topics identified here complement existing country reports,^12^ and may guide and prioritise further research on this topic. This is of particular relevance, given the recent publication of a proposal for a European directive on the rights of cross-border patients, and given a recent report highlighting the heterogeneity with which quality strategies are adopted in the EU member states.^30^ ^31^

## CONCLUSION

This research project obtained information on the viewpoints and priorities of patients, professionals and healthcare-financing bodies regarding the use of healthcare abroad. The information derived from the interviews allowed us to identify many quality requirements for cross-border care as well as issues of organisation and communication, with a potential impact on the quality and safety of cross-border care. Although quality requirements for cross-border care are similar to those of local patients, certain situations exist that require particular attention to the needs of cross-border patients. The findings presented here may help to support further research on patients who cross borders in EU countries, as expressed in the EU Programme of Community Action in the Field of Health (2007–2013) and the recent call for research proposals in this field. It is hoped that our findings will also help to guide future work on developing quality improvement strategies at EU level.

## APPENDIX A

### Topics for further research and quality improvement of cross-border care

#### Perceptions of and information on cross-border care

- What is the perception of patients/citizens about the administrative and financial procedures to obtain cross-border care?
- What is the perception of patients/citizens about the quality of care provided in other EU countries?
- What health systems and hospital information do patients have access to before going abroad for treatment? How does this impact on their willingness to obtain treatment abroad, in particular if cross-border care is offered or recommended by the purchaser?
- Do governments, purchasers and patients use different criteria when selecting/contracting a cross-border care provider?

#### Admission of cross-border patients

- Who checks eligibility for treatment when a foreign EU patient arrives at the hospital? Is this the sole responsibility of hospital administration, or does professional or medical judgement play a role?
- What is the impact of cross-border care provision on the administrative burden of the receiving hospital? Do additional administrative and information requirements make cross-border patients more expensive? How are local co-payments dealt with in administering cross-border patients?
- For planned admissions, what is the patients’ role and duty to present information on the course of the condition (tests, medical record)?

#### Information and communication during the hospital stay

- Once admitted, how do cross-border patients perceive the hospital stay: do they receive information about what is happening to them, and information on scheduled visits and tests? How are their language barriers taken into account?
- Are the strategies used by hospitals to overcome language barriers (such as interpreters or multilingual staff) sufficient to ensure the same level of informed consent, patient information and education for both local and cross-border patients? Are they sufficient to ensure that patients feel that their needs are taken care of, that they are well understood, and that they have an opportunity to discuss their concerns?
- How are differences in informed consent procedures dealt with? How could different legal requirements and patients’ needs be harmonised?
- Are there differences in the way patients from different countries contribute to and collaborate in the care process (expressing themselves, confidence and anxiety)?
- What is the role of cross-border patients’ families and third parties (interpreters, hospital staff speaking foreign languages, case managers, embassies) in the care process?
- How can the hospital meet the catering needs of cross-border patients, given differences in timing and size of meals between countries?

#### Discharge, transfer and follow-up

- What are the effects of previously arranged travel on the length of stay and discharge date of cross-border patients?
- What is the impact of the discharge of cross-border patients on continuity of care? How are different information and legal requirements, language needs and transfers to different types of providers in different countries dealt with?
- Are there any quality and patient safety issues resulting from differences in national and foreign regulations for the quality of care (eg, in the case of transplantation medicine or rehabilitation services)?
- How can patient safety be ensured when providing patients with medication that differs in name, type and doses between countries?
- What are the administrative requirements for arranging medical back-transfer across borders? What are the implications (responsibilities and liabilities) in case of adverse events that occur during medical transport?
